# Factors Affecting Quality of Life Among the Elderly in Long-Term Care Hospitals

**DOI:** 10.1097/jnr.0000000000000413

**Published:** 2020-12-07

**Authors:** Hee-Kyung CHANG, Cho-Rong GIL, Hye-Jin KIM, Han-Ju BEA

**Affiliations:** 1 PhD, RN, Associate Professor, College of Nursing, Gerontological Health Research Center in Institute of Health Sciences, Gyeongsang National University, Jinju, South Korea; 2 MSN, RN, Researcher, College of Nursing, Gerontological Health Research Center in Institute of Health Sciences, Gyeongsang National University, Jinju, South Korea; 3 MPH, RN, Assistant Professor and Researcher, Department of Nursing Science, Kyungsung University, Busan, South Korea; 4 MSN, RN, Assistant Professor, School of Nursing, Yeungnam University College, Nam-gu, Daegu, South Korea.

**Keywords:** depression, long-term care hospital, nurse–patient interaction, older people care, quality of life

## Abstract

**Background:**

There are challenges in sustaining person-centered care in aged care settings. Key related issues of concern such as quality of life among the older people in long-term care hospitals and interactions with nursing staff have been described previously.

**Purpose:**

This study was designed to explore the factors affecting quality of life among older people living in long-term care hospitals in South Korea.

**Methods:**

Older adult patients (*N* = 202) in three long-term care hospitals completed measures of cognitive functions, depression, care dependency, and interactions between nurse and patient and a quality-of-life assessment tool. Univariate analyses were used to examine the relationships among these variables, and a multiple linear regression analysis was used to explore the extent to which these variables predicted quality of life in these patients.

**Results:**

The significant factors associated with quality of life were found to be cognitive functions (*r* = .373, *p* < .001), care dependency (*r* = .350, *p* < .001), and depression (*r* = −.504, *p* < .001). The regression model with depression and care dependency as predictor variables accounted for 25.7% of the variance in quality of life.

**Conclusions/Implications for Practice:**

The correlation found in this study between quality of life and depression and care dependency provides additional evidentiary support for developing and applying nursing interventions that reduce depression and care dependency in older adult populations.

## Introduction

The proportion of the population aged 65 years and above has increased annually, with this proportion (14.9%) surpassing the proportion of youths under 15 years old (12.4%) in 2019. By 2025, South Korea is expected to be a super-aged society, with a population of older people greater than 20% (National Statistical Office, 2019a). With this increase in the older adult population, the number of long-term care hospitals (LTCHs) in Korea increased to 1,577 in 2019, up 202.9% from 2009 (National Statistical Office, 2019b). LTCHs in Korea are medical institutions that provide medical treatment to patients who require care for geriatric or chronic diseases or those who are recovering after surgery or injury (Ministry of Health and Welfare, 2019). Those with health insurance may be admitted to an LTCH by personal request or at the recommendation of a doctor. LTCHs maintain one doctor for every 40 patients and one nurse for every six patients and provide rehabilitation services such as physical therapy and occupational therapy as well as medical services and care services (Song, 2012). As demand for LTCHs continues to increase steadily with population aging, factors such as the quality of life (QoL) of hospitalized older people are expected to become more important than the size or number of hospitals (McDermott et al., 2012).

QoL is a multidimensional concept that includes subjective well-being and happiness and considers personal characteristics as well as physical, psychological, and social aspects (Haas, 1999). QoL is widely considered as an indicator of individual health in explorations of daily living functions and well-being (Bowling, 1995). In particular, QoL in older people is very important, as it is closely related to happiness and the achievement of life goals.

Humans experience a variety of changes in old age such as physical changes because of illness or natural deterioration of physical functions, social changes because of the loss of roles in the home and society, a worsening economic situation, depression, solitude, and even suicidal impulses. Among these changes, the deterioration of physical functions because of aging affects brain function negatively and leads to decreased cognitive functioning, which causes memory problems, disorientation, diminished judgment, and poor comprehension (Gajewski & Falkenstein, 2016). It is known that older people who are socially isolated because of reduced environmental stimulation often have reduced cognitive statuses (Coyle & Dugan, 2012). The decline in cognitive functions in older people makes it difficult for them to perform daily life activities independently, leading to depression and ultimately lowering their QoL (Svantesson et al., 2015).

Depression is one of the most prominent changes in the mental functioning of older people. It is known that older people in LTCHs have a relatively high level of depression because of their disconnection from familiar lifestyles and familiar social relationships (Olsen et al., 2016). However, it is important for older people to assess depression accurately because they often experience depression through physical symptoms such as headaches rather than through feeling depressed (Kamińska et al., 2015). Older people with decreased physical functions in terms of hearing, vision, and physical strength because of aging find it more difficult to perform activities such as eating, dressing, going to the bathroom, moving, and so on. Thus, they tend to rely on others for assistance and care (Meinerding et al., 2018). In consideration of this dependency, as well as the degradation of their human dignity and QoL, recent LTCHs have focused on providing nursing that reflects individual needs to maximize the use of the physical functions of older people and help them sustain an independent way of life (Brownie & Nancarrow, 2013). This suggests that older people's cognitive functions, depression, nursing dependency, and QoL are closely interrelated.

Person-centered care is a concept that has emerged as part of efforts to improve the QoL of older people living in LTCHs. Person-centered care refers to the recognition and practice of respecting the value and abilities of patients to protect their autonomy, self-esteem, and independence and meet their psychological needs (Flesner, 2009). Person-centered care may be effective in improving QoL in the physical, emotional, and social domains (Morgan & Yoder, 2012). Individualized nursing is essential for older people because each older person has unique physical, psychosocial, and mental problems and hence has different needs (Morgan & Yoder, 2012). It has been reported that person-centered care improves QoL and resolves psychological and social problems such as loneliness and depression, which are common in older people, by promoting communication between older people and medical staff (Brownie & Nancarrow, 2013). Therefore, understanding nurse–patient interactions is a prerequisite to providing person-centered care.

The Nurse–Patient Interaction Scale (NPIS), which is an appropriate instrument for understanding the communication and interactions between nurses and patients, includes factors such as having confidence in nurses, experiences of being respected and taken seriously, and satisfaction with communication (Haugan et al., 2012). However, because this scale has not been used in Korea, there is a need for studies and research to measure the interactions between nurses and patients using the NPIS to determine how nurses affect the lives of older people.

Previous studies related to factors affecting the QoL of older people have shown better physical functioning, which is associated with living in the community (De Vries et al., 2012), better cognitive function, and higher social activity (J. Kim et al., 2016), to be related to higher QoL. Older people in nursing homes with higher QoL were found to have lower levels of depression and better performance in activities of daily living (Barca et al., 2011). In South Korea, LTCHs provide not only medical treatment and care but also a place to live until death (Yi & Lee, 2015). In addition, as the cost burden is relatively low and the hospitalization procedure is simple, the number of older people hospitalized in LTCHs will likely continue to increase. However, there is insufficient research on the QoL of older people living in LTCHs.

Therefore, to study the QoL of older people who are hospitalized in LTCHs, it is necessary to adopt an integrated approach that includes physical factors such as care dependence, emotional factors such as depression, and social factors such as interactions. Therefore, this study was designed to identify the factors affecting the QoL of older people hospitalized in LTCHs as a reference for the development of nursing interventions that effectively improve the QoL of older LTCH residents.

### Aims

The purpose of this study was to explore the QoL and related factors (cognitive functions, depression, care dependency, and nurse–patient interactions) of older people living in LTCHs and to provide necessary data for the development of nursing interventions to improve the QoL of people in this vulnerable population.

## Methods

### Design

This was a descriptive study of the QoL of older people hospitalized in LTCHs and related factors.

### Settings and Sample

Ethical approval was received from the Gyeongsang University Research Board of Ethics (Approval No. GIRB-A18-Y-0030), and approval to access and use medical data containing personal information was obtained from the LTCHs in Suncheon, Gwangyang. To select the participants for this study, a nurse who was currently working at an LTCH recommended three LTCHs with capacities of over 200 patients each. Convenience sampling was used in this study. LTCHs A and B are located in Suncheon City, and LTCH C is located in Gwangyang City, with 245, 250, and 299 beds, respectively. They all received a first grade in the nursing grade evaluation conducted by the Ministry of Health and Welfare, which considers financial factors, programs, services, and resident rights.

The participants in this study (*N* = 202) were older people who had been admitted to one of the three participating LTCHs. All met the definition of an older person (> 65 years old) used by the United Nations and the Korean Welfare Law. First, to receive the approval of the institutions to collect data, we explained the purpose of the study to the administrators and obtained permission to proceed. Older people who were able to communicate and could respond to questions were recommended by the nurse working in LTCHs. After explaining the study purpose and obtaining informed consent, we collected information from the participants and their guardians. The inclusion criteria were as follows: (a) age of 65 years or older and were admitted to an LTCH, (b) ability to communicate sufficiently with the nurse, and (c) agreement to participate in the study and submit written consent (both participant and guardian). Those who had abused drugs or alcohol and those with severe intellectual disabilities who were unable to communicate were excluded.

The sample size was calculated using the G*Power 3.1.2 program. On the basis of an effect size of *d* = .15, a significance level of α = .05, and a power of (1 − β) = .95, the number of samples required for a multiple regression analysis, including five independent variables, was estimated to be 138. Considering attrition because of mortality, a sample of 202 participants was recruited. Sixty-seven completed questionnaires were collected at LTCH A, 82 were collected at LTCH B, and 53 were collected at LTCH C.

### Measures

Cognitive impairment was assessed using the Korean version of the Mini-Mental State Examination (MMSE-K; Kwon & Park, 1989). This tool was slightly modified from the original tool, the MMSE, based on the high rate of uneducated older people in South Korea. The MMSE-K consists of 12 questions: one item on time orientation (5 points), two items on location orientation (5 points), one item on memory registration (3 points), one item on memory recall (3 points), one item on focus and calculation (5 points), four items on language function (7 points), and one item on understanding and judgment (2 points), for 30 points in total. In cases in which a participant had no formal education, 3 additional points were allocated to the time orientation, attention, and language items. Those scoring between 24 and 30 points were categorized as having no cognitive impairment, those scoring between 18 and 23 points were categorized as having mild cognitive impairment, and those scoring 17 points or less were categorized as having severe cognitive impairment. MMSE-K is a tool with high reliability and validity, with a test–retest reliability of .89 and interscorer reliability of .82 at the time of development.

Depression was assessed using a Korean translation of the Short Geriatric Depression Scale developed by Sheikh and Yesavage (1986) as a simplified 15-item version of the Geriatric Depression Scale (GDS), which was developed by Cho et al. (1999). Answers take the form of “yes” or “no,” and the total possible score is 15. The Short Geriatric Depression Scale showed satisfactory sensitivity and specificity when applied to the cutoff score of 8 in a study of neuropsychiatric ward patients (Cho et al., 1999). Therefore, the cutoff point used in the current study was 8, with a score of > 8 indicating depression. The Cronbach's α of this scale was .94 in Sheikh and Yesavage (1986) and Kuder–Richardson Formula 20 was .84 in this study.

Care dependency was assessed using the Korean version of the Care Dependency Scale (CDS-Korean version), which was modified in 2000 by Kim (E. J. Kim, 2000) from the original CDS-UK developed by Dijkstra et al. (2000). The CDS is a scale derived from observed behavior that is relatively easy for nurses and caregivers to measure. In this study, the surveyor observed the participants and filled out the questionnaire, which was previously confirmed by nurses who care for older people. The CDS consists of 15 items rated on a 5-point Likert scale, ranging from 1 (*completely dependent*) to 5 (*effectively independent*). Scale items address issues such as “eating/drinking,” “communication,” and “recreational activities.” Total possible scores range from 15 to 70, with higher scores indicating lower care dependency. The Cronbach's α of this scale was .93 in the study of Kim and .96 in this study.

Interactions between nurses and patients were assessed using the NPIS, which was developed by Haugan et al. (2012). It consists of 14 items rated on a 10-point Likert scale, ranging from 1 (*not at all*) to 10 (*very much*). Total possible scores range from 14 to 140, with higher scores indicating better nurse–patient interaction. Scale items address statements such as “The nurses make all possible efforts to relieve my medical problems” and “I often am hurt or saddened by how the nurses interact with me.” Although the NPIS has not been used in South Korea, this scale is widely used in Europe and the United States to describe interactions between patients and nursing staff in long-term care settings. The NPIS has shown good content validity and reliability among nursing home patients (Haugan et al., 2012). After approval from the tool's original developer and before data collection, the questionnaire was reviewed by an expert with years of practical experience in an LTCH and by a professor fluent in both English and Korean, who back-translated the NPIS into English and confirmed that no significant differences existed between the original English and Korean versions. The Cronbach's α of this scale was .92 in the study of Haugan et al. and was .92 in this study.

QoL was assessed using the Korean version of the Quality of Life-Alzheimer's Disease (KQoL-AD; Shin, 2006). The KQoL-AD was modified to fit the QoL-AD developed by Logsdon et al. (1999), which consists of 13 items rated on a 4-point Likert scale, ranging from 1 (*bad*) to 4 (*very good*). Total possible scores ranged from 13 to 52, with lower scores indicating worse QoL. Scale items address issues such as physical health, family, and ability to do things for fun. KQoL-AD is a tool for evaluating QoL in patients with dementia that has excellent content validity and test–retest reliability. As KQoL-AD is not affected by cognitive function or the dementia stage, this scale has been used on patients with non-dementia-related cognitive impairments as well as on patients with dementia (H. J. Kim et al., 2008). KQoL-AD may be used to evaluate the QoL in patients using self-reported data as well as using responses provided by caregivers. In this study, those participants who expressed experiencing difficulties in filling out this scale were permitted to have their legal agents complete the scale on their behalf. The Cronbach's α of the KQoL-AD was .88 in the study of Shin and was .88 in this study.

### Data Collection

Data were collected for this study from July to August 2018. Two research assistants and three nurses trained in administering the survey for this study informed each of the participants and their legal guardians regarding the study purpose and explained that they could withdraw at any time. In addition, it was clearly stated in the questionnaire that all information from the survey would only be used for the purpose of this study and that all personal and medical information would be anonymized.

One-on-one interviews and observations with study participants were conducted by the research assistants. The research assistants read the questionnaire aloud and recorded the participant's responses. Four researchers were responsible for planning and overseeing the entire study. To minimize the influence of the researchers, the researchers informed the two research assistants, who were nursing students, about the study purpose, the precautions to take when collecting data, and measurement tool contents and methods. The goal of this precautionary activity was to maintain consistency in the methods and attitudes of the research assistants. After listening to all of the explanations, the participants and legal guardians who agreed to participate in the study were interviewed. General characteristics that were not definitively known by the participants (e.g., cost payer) were confirmed through legal guardians or medical records. The data collected through research assistant observations such as CDS were checked by three nurses who were trained in performing questionnaires. The survey took 20–30 minutes to complete, and the data were collected by four researchers on the same day. Two hundred two questionnaires were distributed, and all 202 were completed and used in the final analysis.

### Data Analyses

SPSS Version 22.0 (IBM, Inc., Armonk, NY, USA) was used for statistical analyses. The characteristics, cognitive functions, depression, care dependency, nurse–patient interaction, and QoL of the participants were analyzed using descriptive analysis. Cronbach's alpha reliability analyses were conducted on data related to cognitive function, depression, care dependency, nurse–patient interaction, and QoL. The correlations between cognitive functions, depression, care dependency, nurse–patient interaction, and QoL were analyzed using correlation analyses and multiple regression analyses. The significance level was set at .05.

## Results

### Differences in the Mean Values of Quality of Life According to the Characteristics

The sample consisted of 202 participants, of whom 145 (71.8%) were female and 57 (28.2%) were male (Table 1). The age of participants ranged from 65 to 96 years, with a mean of 80.61 years. Slightly over half (56.4%) of the participants had no formal education, 71.8% of cost payers were the participants' children, 43.6% of the participants had lived in their current LTCH for over 2 years, and 18.3% of the participants were visited by family members less than once per month. Two thirds (66.3%) of the participants had severe cognitive impairment, and 63.9% had a GDS score over 8. Number of family visits (*F* = 6.90, *p* < .001), cognitive function (*F* = 10.70, *p* < .001), and depression tendencies (*t* = 6.94, *p* < .001) were found to have statistically significant impacts on the QoL of participants.

**Table 1.** Differences in Mean Values of Quality of Life, by Personal Characteristics (*N* = 202)

Characteristic	*n*	%	Quality of Life				
			Mean	*SD*	*t/F*	*p*	Scheffe
Gender					−0.98	.328	
Male	57	28.2	26.68	6.00			
Female	145	71.8	27.58	5.77			
Age (years; *M* and *SD*)	80.61	4.77			1.02	.385	
≤ 69	14	6.9	27.07	5.54			
70–79	75	37.1	28.00	5.44			
80–89	92	45.6	27.23	6.16			
> 89	21	10.4	25.52	5.89			
Education					0.49	.741	
Elementary school	69	34.2	27.81	6.11			
Middle school	8	4.0	28.88	10.41			
High school	9	4.4	28.22	6.36			
College or more	2	1.0	27.00	1.41			
No formal education	114	56.4	26.86	5.28			
Cost payer					2.22	.087	
Self	35	17.3	26.11	6.53			
Spouse	8	4.0	27.50	8.32			
Children	145	71.8	27.90	5.56			
Governmental subsidies	14	6.9	24.36	4.22			
Length of stay (years)					1.61	.203	
< 1	68	33.6	27.54	5.62			
1–2	46	22.8	28.43	5.75			
> 2	88	43.6	26.58	6.00			
Family visits (per month)					6.90	< .001	① > ③; ② > ③
① Usually (3 times and over)	81	40.1	28.47	6.09			
② Often (1–2 times)	84	41.6	27.55	4.99			
③ Seldom (< 1 time)	37	18.3	24.32	6.15			
MMSE-K					10.70	< .001	① > ③; ② > ③
① No cognitive impairment	32	15.9	30.38	4.92			
② Mild cognitive impairment	36	17.8	29.36	5.29			
③ Severe cognitive impairment	134	66.3	26.05	5.78			
GDS (score)					6.94	< .001	① > ②
① ≤ 8	96	36.1	30.02	5.94			
② > 8	106	63.9	24.89	4.53			

***Note.*** MMSE-K = Korean version of the Mini-Mental State Examination for dementia screening; GDS = Geriatric Depression Scale.

### Descriptive Statistics of the Care Dependency Scale and Quality of Life

The average item score on the CDS was 3.26 (*SD* = 0.84), with the communication score (3.76 ± 1.13) earning the highest average score and the recreational activities score (2.85 ± 1.10) earning the lowest (Table 2). The average item score on the KQoL-AD was 2.10 (*SD* = 0.03), with the family relationship score (2.43 ± 0.68) earning the highest average score and the physical health score (1.90 ± 0.74) earning the lowest (Table 3).

**Table 2.** Care Dependency Scale (*N* = 202)

Item	Mean	*SD*
Eating/drinking	3.48	1.02
Incontinence	3.51	1.32
Body posture	3.40	1.12
Mobility	2.96	1.22
Day/night pattern	3.59	1.10
Getting (un)dressed	3.25	1.12
Body temperature	3.36	1.11
Hygiene	3.00	1.12
Avoidance of danger	3.16	1.01
Communication	3.76	1.13
Contact with others	3.26	1.13
Sense of rules/values	3.58	1.20
Daily activities	3.21	1.23
Recreational activities	2.85	1.10
Learning ability	2.86	1.13
Total score, mean and *SD*	49.23	13.71

***Note.*** Range of each item score from 1 to 5.

**Table 3.** Korean Version of the Quality of Life-Alzheimer's Disease Scale (*N* = 202)

Item	Mean	*SD*
Physical health	1.90	0.74
Energy	1.95	0.71
Mood	2.23	0.65
Living situation	2.34	0.67
Memory	2.05	0.74
Family	2.43	0.68
Marriage	2.21	0.74
Friends	2.31	0.72
Self	2.12	0.66
Ability to do chores	1.90	0.76
Ability to do things for fun	1.93	0.72
Money	1.93	0.65
Life as a whole	2.04	0.68
Total score, mean and *SD*	27.33	5.83

***Note.*** Range of each item score: 1–4.

### Correlations Among Cognitive Functions, Depression, Care Dependency, Interaction Between Nurse and Patient, and Quality of Life

The correlations among cognitive function, depression, care dependency, interactions with nurses, and QoL were studied using correlation analysis (Table 4). The results indicate a positive correlation between cognitive functioning and QoL (*r* = .373, *p* < .001) and care dependency (*r* = .350, *p* < .001), respectively; a negative correlation between depression and QoL (*r* = −.504, *p* < .001); and a positive correlation between the NPIS and care dependency (*r* = .235, *p* < .001).

**Table 4.** Correlations Among MMSE-K, GDS, Care Dependency, NPIS, and Quality of Life (*N* = 202)

Variable	MMSE-K	GDS	CDS	NPIS	Quality of Life
MMSE-K	1				
GDS	−.273***	1			
CDS	.570***	−.265***	1		
NPIS	.019	−.019	.235***	1	
Quality of life	.373***	−.504***	.350***	.053	1

***Note.*** MMSE-K = Korean version of the Mini-Mental State Examination for dementia screening; GDS = Geriatric Depression Scale; CDS = Care Dependency Scale; NPIS = Nurse–Patient Interaction Scale; Quality of life = Korean version of the Quality of Life-Alzheimer's Disease Scale.

***p < .001.

### Factors Contributing to Quality of Life

The results of the multiple regression analysis on the factors affecting QoL in older people are shown in Table 5. GDS and CDS was found to be statistically significantly related to QoL (*F* = 35.838, *p* < .001) at a power of prediction of 26.5% (*R* = .515, *R*^2^ = .265).

**Table 5.** Multiple Regression Analysis of Quality of Life With the Other Variables (*N* = 202)

Source Variance	*df*	*SS*	*MS*	*F*	*p*
Model 1 ^a^					
Regression	1	7.857	7.857	48.192	< .001
Residual	200	32.607	0.163		
Total	201	40.464			
Model 2 ^b^					
Regression	2	10.715	5.358	35.838	< .001
Residual	199	29.749	0.149		
Total	201	40.464			

***Note.*** GDS = Geriatric Depression Scale; CDS = Care Dependency Scale.

^a^*R* = .441, *R*^2^ = .194, *SE* = .40378. ^b^*R* = .515, *R*^2^ = .265, *SE* = .38664.

## Discussion

In this study, the QoL of older people in LTCHs and related factors were explored to provide basic data on nursing interventions that may be used to develop interventions to improve QoL in this population.

As shown in Table 1, as women in South Korea have a longer life expectancy than men and as most older women do not have jobs, the risk of social isolation and poverty among older women whose husbands have passed away is high, leading to higher incidences of children taking joint responsibility for parental financial and psychological support (Shin & Park, 2018). Moreover, care by professional staff is required for older people with cognitive impairment. To resolve these problems, older people and their families in South Korea tend to choose LTCHs, which are cheaper than general hospitals and allow the integration of long-term hospitalization with daily life. However, it is believed that older people experience depression because they are disconnected from social support systems such as family and/or close friends, experience decreased social activity, and experience a monotonous daily life after moving into an LTCH.

The mean QoL score for participants in this study was 2.10 ± 0.03, which is slightly below median. This compares with the score of 31.82 ± 7.18 for community-dwelling older people registered at the Dementia Prevention Center (Park & Ahn, 2017). It is thought that the disconnection from support systems, the loss of social life, feelings of abandonment by their family, the stress of adapting to a new environment, and the monotonous daily life in LTCHs all affect QoL.

The mean care dependency score for participants in this study was 3.26 ± 0.84, which is higher than the mean 2.94 ± 0.82 score for 103 patients with stroke (I. Kim, 2018), indicating that older people in LTCHs are more independent than poststroke patients. This is thought to be because of the relatively high severity of poststroke patients' conditions compared with older adults, who often require chronic disease or geriatric disease treatment/management. Unlike adolescents and adults in general, leisure and physical activities for older people are known to play an important role in maintaining health and energy in old age (Shimada et al., 2016). High levels of physical activity in older people have been shown to associate significantly with cognitive function in previous studies (Grimaud et al., 2017; Lin et al., 2012). In particular, after a 6-month aerobic exercise intervention, an increase in hippocampal volume was observed in older people with mild cognitive impairment (Ten Brinke et al., 2015). Therefore, it is possible to improve the independence of older people by applying pleasureful recreational activities and exercise together to stimulate cognitive function during leisure time (Tyndall et al., 2018).

The nurse–patient interaction score for older people in LTCHs was 7.55 ± 1.54. In Norway, the NPIS of nursing home patients without cognitive impairment was reported as 8.13 ± 1.63 (Haugan et al., 2016). The difference was considered to be because of the relatively low cognitive functioning of the participants in this study. It is known that improvements in communication between older people and medical staff improve the former's QoL by helping them deal with problems common to older people such as loneliness and depression (Sagong & Lee, 2016). Thus, interventions based on interactions such as storytelling (Astell et al., 2010) should be implemented to help older people build healthy relationships with other older people or healthcare providers and experience high-quality interactions.

In this study, a significant difference in the QoL was found based on number of family visits (*F* = 6.90, *p <* .001), cognitive functions (*F* = 10.70, *p* < .001), and depression tendencies (*t* = 6.94, *p* < .001). This result supports the findings in previous studies that associated higher family visit frequencies with higher cognitive functioning and lower levels of depression with higher QoL (Barca et al., 2011; Gräske et al., 2015). Because of the traditional values of filial piety and familyism, it is common in South Korea for children to take care of their aging parents. For Korean older people, family is a basic requirement for successful aging as well as the most important source of support. Therefore, a nurse should assess the degree of family visitation together with the patient's perception of the level of family support and maintain close relationships via phone calls and messages. It is also necessary to create an institutional environment in which the family feels comfortable and is able to interact freely with their family member.

Decreased cognitive functioning affects sense of balance, making independent physical activity difficult and reducing QoL in older people (Shin et al., 2011). Physical activity is used as a helpful therapeutic care strategy to delay the loss of functional independence in older people. In particular, regular exercise is known to play an important role in increasing cerebral blood flow and neurotransmitter release, enhancing muscle flexibility and balance, and improving cognitive functioning (Gligoroska & Manchevska, 2012). Therefore, it is desirable to provide an exercise intervention program that maintains and enhances cognitive functioning and daily performance abilities in older people to improve QoL.

Depression in older people not only lowers physical functioning but, if neglected, may also cause cognitive dysfunction and dementia (Demnitz et al., 2016). Moreover, depression may threaten the health of older people because of its association with a higher risk of suicide. However, early detection of depression and proper medication or psychotherapy may help relieve symptoms and improve QoL. Therefore, active prevention, early detection, and appropriate treatment are very important. LTCH nurses are responsible to provide depression-related prevention education, early detection, and treatment. Thus, training in diagnosing depression should be provided to all healthcare providers who care for older people.

Depression and nursing dependence were found in this study to be predictor variables of QoL, explaining 26.5% of the variance in QoL. This is similar to a previous study that found depression, behavior, and degree of care needed for daily life to be related to the QoL of 181 people aged 65 years or older who were admitted to LTCHs (Ok & Lee, 2017). Therefore, we should pay attention to depression in older people hospitalized in LTCHs and try to improve their QoL using appropriate diagnosis and treatment. Furthermore, nurses should create an atmosphere in which older people may participate proactively in decision making and increase their independence and interactivity using health checks and daily living ability reinforcement programs.

This study adds to the scholarly knowledge by exploring the QoL of older people who were admitted to the unique environment of an LTCH, which is an institution midway between traditional hospital and nursing home settings. LTCHs provide care that addresses the physical, emotional, and social domains of patients. However, nurse–patient interactions, which were expected to relate significantly to QoL, were found to relate only insignificantly. Possibly because nurses submitted the surveys on behalf of the senior participants who had low cognitive functions. In a previous study (Haugan et al., 2012), the NPIS was related to QoL for nursing home patients with normal cognitive functioning. Thus, this issue should be reexamined and confirmed in future studies. Finally, the results of this study should be interpreted cautiously because of its use of a convenience sample of older people who were admitted to three LTCHs in two areas of South Korea.

### Conclusions

QoL is a critical issue for the rising population of older adults living in LTCHs in South Korea. The purpose of this study was to investigate the effects of general characteristics, cognitive functioning, care dependency, depression, and nurse–patient interactions on the QoL of older people admitted to LTCHs and to suggest improvements. On the basis of the results of this study, the development and implementation of a nursing intervention method that lowers depression and care dependency in older people living in LTCHs may improve their QoL.

## References

[bib1] Astell A. J. Ellis M. P. Bernardi L. Alm N. Dye R. Gowans G., & Campbell J. (2010). Using a touch screen computer to support relationships between people with dementia and caregivers. *Interacting with Computers*, 22(4), 267–275. 10.1016/j.intcom.2010.03.003

[bib2] Barca M. L. Engedal K. Laks J., & Selbæk G. (2011). Quality of life among elderly patients with dementia in institutions. *Dementia and Geriatric Cognitive Disorders*, 31(6), 435–442. 10.1159/000328969 21757909

[bib3] Bowling A. (1995). What things are important in people's lives? A survey of the public's judgements to inform scales of health related quality of life. *Social Science & Medicine*, 41(10), 1447–1462. 10.1016/0277-9536 (95)00113-L 8560313

[bib4] Brownie S., & Nancarrow S. (2013). Effects of person-centered care on residents and staff in aged-care facilities: A systematic review. *Clinical Interventions in Aging*, 8, 1–10. 10.2147/CIA.S38589 23319855 PMC3540911

[bib5] Cho M. J. Bae J. N. Suh G. H. Hahm B. J. Kim J. K. Lee D. W., & Kang M. H. (1999). Validation of Geriatric Depression Scale, Korean version (GDS) in the assessment of DSM-III-R major depression. *Journal of Korean Neuropsychiatric Association*, 38(1), 48–63 (Original work published in Korean).

[bib6] Coyle C. E., & Dugan E. (2012). Social isolation, loneliness and health among older adults. *Journal of Aging and Health*, 24(8), 1346–1363. 10.1177/0898264312460275 23006425

[bib7] De Vries N. M. Van Ravensberg C. D. Hobbelen J. S. M. Rikkert M. O. Staal J. B., & Nijhuis-Van der Sanden M. W. G. (2012). Effects of physical exercise therapy on mobility, physical functioning, physical activity and quality of life in community-dwelling older adults with impaired mobility, physical disability and/or multi-morbidity: A meta-analysis. *Ageing Research Reviews*, 11(1), 136–149. 10.1016/j.arr.2011.11.002 22101330

[bib8] Demnitz N. Esser P. Dawes H. Valkanova V. Johansen-Berg H. Ebmeier K. P., & Sexton C. (2016). A systematic review and meta-analysis of cross-sectional studies examining the relationship between mobility and cognition in healthy older adults. *Gait & Posture*, 50, 164–174. 10.1016/j.gaitpost.2016.08.028 27621086 PMC5081060

[bib9] Dijkstra A. Brown L. Havens B. Romeren T. I. Zanotti R. Dassen T., & Van Den Heuvel W. (2000). An international psychometric testing of the care dependency scale. *Journal of Advanced Nursing*, 31(4), 944–952. 10.1046/j.1365-2648.2000.01354.x 10759991

[bib10] Flesner M. K. (2009). Person-centered care and organizational culture in long-term care. *Journal of Nursing Care Quality*, 24(4), 273–276. 10.1097/NCQ.0b013e3181b3e669 19755877

[bib11] Gajewski P. D., & Falkenstein M. (2016). Physical activity and neurocognitive functioning in aging—A condensed updated review. *European Review of Aging and Physical Activity*, 13(1), 1–7. 10.1186/s11556-016-0161-3 26865880 PMC4748322

[bib12] Gligoroska J. P., & Manchevska S. (2012). The effect of physical activity on cognition–physiological mechanisms. *Materia Socio-medica*, 24(3), 198–202. 10.5455/msm.2012.24.198-202 23678325 PMC3633396

[bib13] Gräske J. Meyer S. Worch A., & Wolf-Ostermann K. (2015). Family visits in shared-housing arrangements for residents with dementia–A cross-sectional study on the impact on residents' quality of life. *BMC Geriatrics*, 15(1), Article 14 10.1186/s12877-015-0012-5 25868401 PMC4347913

[bib14] Grimaud E. Taconnat L., & Clarys D. (2017). Cognitive stimulation in healthy older adults: A cognitive stimulation program using leisure activities compared to a conventional cognitive stimulation program. *Geriatrie et Psychologie Neuropsychiatrie du Vieillissement*, 15(2), 214–223. 10.1684/pnv.2017.0669 (Original work published in French) 28625942

[bib15] Haas B. K. (1999). A multidisciplinary concept analysis of quality of life. *Western Journal of Nursing Research*, 21(6), 728–742. 10.1177/01939459922044153 11512210

[bib16] Haugan G. Moksnes U. K., & Løhre A. (2016). Intrapersonal self-transcendence, meaning-in-life and nurse–patient interaction: Powerful assets for quality of life in cognitively intact nursing-home patients. *Scandinavian Journal of Caring Sciences*, 30(4), 790–801. 10.1111/scs.12307 26917325

[bib17] Haugan G. Rannestad T. Hanssen B., & Espnes G. A. (2012). Self-transcendence and nurse–patient interaction in cognitively intact nursing home patients. *Journal of Clinical Nursing*, 21(23–24), 3429–3441. 10.1111/j.1365-2702.2012.04217.x 23145515

[bib18] Kamińska M. Brodowski J., & Karakiewicz B. (2015). Fall risk factors in community-dwelling elderly depending on their physical function, cognitive status and symptoms of depression. *International Journal of Environmental Research and Public Health*, 12(4), 3406–3416. 10.3390/ijerph120403406 25811765 PMC4410192

[bib19] Kim E. J. (2000). Psychometric testing of the care dependency scale for the institutionalized Korean demented patients. *Journal of Korean Academy of Psychiatric and Mental Health Nursing*, 9(3), 303–314. (Original work published in Korean).

[bib20] Kim H. J. Moon S. Y. Kim S. Y., & Han S.-H. (2008). Assessment of the quality of life in patients with Alzheimer's disease. *Journal of the Korean Neurological Association*, 26(4), 308–313. (Original work published in Korean).

[bib21] Kim I. (2018). Care dependency of post-stroke patients and its affecting factors. *Korean Journal of Rehabilitation Nursing*, 21(2), 100–109. (Original work published in Korean).

[bib22] Kim J. Song R. Kim K. W., & Kim J. L. (2016). Effect of cognitive function, social activity participation and social support on quality of life of community-dwelling elderly. *Journal of Korean Geriatric Psychiatry*, 20(1), 25–32. 10.0000/jkgp.2016.20.1.25 (Original work published in Korean)

[bib23] Kwon Y. C., & Park J. H. (1989). Korean version of Mini-Mental State Examination (MMSE-K). *Journal of the Korean Neuropsychiatric Association*, 28(1), 125–135. (Original work published in Korean).

[bib24] Lin F. Friedman E. Quinn J. Chen D. G. D., & Mapstone M. (2012). Effect of leisure activities on inflammation and cognitive function in an aging sample. *Archives of Gerontology and Geriatrics*, 54(3), 398–404. 10.1016/j.archger.2012.02.002 PMC3331938 22377120

[bib25] Logsdon R. G. Gibbons L. E. McCurry S. M., & Teri L. (1999). Quality of life in Alzheimer's disease: Patient and caregiver reports. *Journal of Mental Health and Aging*, 5(1), 21–32.

[bib26] McDermott C. Coppin R. Little P., & Leydon G. (2012). Hospital admissions from nursing homes: A qualitative study of GP decision making. *British Journal of General Practice*, 62(601), e538–e545. 10.3399/bjgp12X653589 PMC3404331 22867677

[bib27] Meinerding M. DeFeis B. Sunderaraman P. Azar M. Lawless S. Perez-Vivaldo C. Gu Y. Stern Y., & Cosentino S. (2018). Assessing dependency in a multiethnic community cohort of individuals with Alzheimer's disease. *Innovation in Aging*, 2(1), 1–7. 10.1093/geroni/igy011 29795795 PMC5954614

[bib28] Ministry of Health and Welfare (2019). *Medical law*. Retrieved November 23, 2019, from http://www.law.go.kr/%EB%B2%95%EB%A0%B9/%EC%9D%98%EB%A3%8C%EB%B2%95

[bib29] Morgan S., & Yoder L. H. (2012). A concept analysis of person-centered care. *Journal of Holistic Nursing*, 30(1), 6–15. 10.1177/0898010111412189 21772048

[bib30] National Statistical Office (2019a). *2019 elderly statistics*. Retrieved September 28, 2020, from http://kostat.go.kr/portal/korea/kor_nw/1/6/1/index.board?bmode=read&bSeq=&aSeq=377701&pageNo=2&rowNum=10&navCount=10&currPg=&searchInfo=&sTarget=title&sTxt=

[bib31] National Statistical Office (2019b). *Hospital present conditions based on cities and provinces*. Retrieved September 28, 2020, from http://kosis.kr/statHtml/statHtml.do?orgId=354&tblId=DT_MIRE01#

[bib32] Ok E. M., & Lee J. Y. (2017). A study on influential factors to the quality of life in geriatric hospital elderly inpatient. *Journal of Wholistic Nursing Science*, 10(1), 1–12. (Original work published in Korean).

[bib33] Olsen C. Pedersen I. Bergland A. Enders-Slegers M. J. Patil G., & Ihlebaek C. (2016). Effect of animal-assisted interventions on depression, agitation and quality of life in nursing home residents suffering from cognitive impairment or dementia: A cluster randomized controlled trial. *International Journal of Geriatric Psychiatry*, 31(12), 1312–1321. 10.1002/gps.4436 26807956

[bib34] Park S. H., & Ahn S. N. (2017). The effect of dual task program on reducing the risk of dementia in older adults. *Research Journal of Pharmacy and Technology*, 10(7), 2255–2259. 10.5958/0974-360X.2017.00399.7 (Original work published in Korean)

[bib35] Sagong H., & Lee G. E. (2016). Person-centered care and nursing service quality of nurses in long-term care hospitals. *Journal of Korean Academy of Community Health Nursing*, 27(4), 309–318. 10.12799/jkachn.2016.27.4.309

[bib36] Sheikh J. I., & Yesavage J. A. (1986). Geriatric Depression Scale (GDS): Recent evidence and development of a shorter version. *Clinical Gerontologist: The Journal of Aging and Mental Health*, 5(1–2), 165–173. 10.1300/J018v05n01_09

[bib37] ShimadaH.MakizakoH.LeeS.DoiT.TsutsumimotoK.HaradaK.HottaR.BaeS.HaradaN. K., & SuzukiT. (2016). Impact of cognitive frailty on daily activities in older persons. *The Journal of Nutrition, Health & Aging*, 20(7), 729–735. 10.1007/s12603-016-0685-227499306

[bib38] Shin B. M. Han S. J. Jung J. H. Kim J. E., & Fregni F. (2011). Effect of mild cognitive impairment on balance. *Journal of the Neurological Sciences*, 305(1-2), 121–125. (Original work published in Korean). 21420690 10.1016/j.jns.2011.02.031

[bib39] Shin H. Y. (2006). A preliminary study on the Korean version of quality of life-Alzheimer's disease scale in community-dwelling elderly with dementia. *Journal of Preventive Medicine and Public Health*, 39(3), 243–248. (Original work published in Korean). 16764499

[bib40] Shin S. H., & Park J. S. (2018). A structural equation model of quality of life in nursing home residents. *Journal of Korean Gerontological Nursing*, 20(3), 193–203. 10.17079/jkgn.2018.20.3.193 (Original work published in Korean)

[bib41] Song H. J. (2012). Long-term care hospital systems in developed countries and the implications for Korea. *Journal of the Korean Geriatrics Society*, 16(3), 114–120. (Original work published in Korean).

[bib42] Svantesson U. Jones J. Wolbert K., & Alricsson M. (2015). Impact of physical activity on the self-perceived quality of life in non-frail older adults. *Journal of Clinical Medicine Research*, 7(8), 585–593. 10.14740/jocmr2021w 26124903 PMC4471744

[bib43] Ten Brinke L. F. Bolandzadeh N. Nagamatsu L. S. Hsu C. L. Davis J. C. Miran-Khan K., & Liu-Ambrose T. (2015). Aerobic exercise increases hippocampal volume in older women with probable mild cognitive impairment: A 6-month randomised controlled trial. *British Journal of Sports Medicine*, 49(4), 248–254. 10.1136/bjsports-2013-093184 24711660 PMC4508129

[bib44] Tyndall A. V. Clark C. M. Anderson T. J. Hogan D. B. Hill M. D. Longman R. S., & Poulin M. J. (2018). Protective effects of exercise on cognition and brain health in older adults. *Exercise and Sport Sciences Reviews*, 46(4), 215–223. 10.1249/JES.0000000000000161 30001269

[bib45] Yi M. J., & Lee J. S. (2015). Nurse's experiences of the death of patients in geriatric hospitals. *Journal of Korean Academy of Nursing*, 45(4), 513–522. 10.4040/jkan.2015.45.4.513 26364526

